# An advanced human *in vitro* co-culture model for translocation studies across the placental barrier

**DOI:** 10.1038/s41598-018-23410-6

**Published:** 2018-03-29

**Authors:** Leonie Aengenheister, Kerda Keevend, Carina Muoth, René Schönenberger, Liliane Diener, Peter Wick, Tina Buerki-Thurnherr

**Affiliations:** 1 Particles-Biology Interactions, Empa, Swiss Federal Laboratories for Materials Science and Technology, Lerchenfeldstrasse 5, 9014 St. Gallen, Switzerland; 2 UTOX, EAWAG, Swiss Federal Institute of Aquatic Science and Technology, Ueberlandstrasse 133, 8600 Dübendorf, Switzerland

## Abstract

Although various drugs, environmental pollutants and nanoparticles (NP) can cross the human placental barrier and may harm the developing fetus, knowledge on predictive placental transfer rates and the underlying transport pathways is mostly lacking. Current available *in vitro* placental transfer models are often inappropriate for translocation studies of macromolecules or NPs and do not consider barrier function of placental endothelial cells (EC). Therefore, we developed a human placental *in vitro* co-culture transfer model with tight layers of trophoblasts (BeWo b30) and placental microvascular ECs (HPEC-A2) on a low-absorbing, 3 µm porous membrane. Translocation studies with four model substances and two polystyrene (PS) NPs across the individual and co-culture layers revealed that for most of these compounds, the trophoblast and the EC layer both demonstrate similar, but not additive, retention capacity. Only the paracellular marker Na-F was substantially more retained by the BeWo layer. Furthermore, simple shaking, which is often applied to mimic placental perfusion, did not alter translocation kinetics compared to static exposure. In conclusion, we developed a novel placental co-culture model, which provides predictive values for translocation of a broad variety of molecules and NPs and enables valuable mechanistic investigations on cell type-specific placental barrier function.

## Introduction

The placenta acts as an interface between the fetus and the expecting mother. This transient organ enables and supports pregnancy by e.g. producing essential hormones, allowing the exchange of gases, nutrients and waste products and forms an essential barrier to protect the growing fetus from exposure to xenobiotics. However, it has been recognized that various drugs, environmental pollutants and even certain nanoparticles (NP) are able to cross the placental barrier^1–6^.

At least since the thalidomide debacle in the 1960’s^7^, research on drug transfer from the maternal to the fetal side and their potential teratogenic effects has been intensified. Nevertheless, the pregnant woman is still considered a drug orphan, and medication development, evaluation and safety trials in pregnancy are markedly under-represented^8^. Despite the fact that comprehensive drug safety and efficacy data are often lacking, the use of drugs in pregnancy is widespread^9,10^. Drugs that are used to treat acute or chronic diseases of the expecting mother could jeopardize pregnancy and the health of the unborn child^11–14^. In contrast, prenatal drug treatment of fetal diseases or placental complications could elicit adverse side effects in the mother^15,16^. Thus safe and highly specific therapeutic strategies to treat the mother, the fetus or placental conditions without off-target effects are of great interest. A highly promising strategy to enhance targeted therapy and avoid undesired side effects is the use of NPs as drug carriers^17–20^. However, the impact of NP properties and surface modifications on placental uptake, accumulation and translocation as well as the underlying transport mechanisms are not yet clearly understood^21–23^.

Animals, mainly rodents, can provide information on placental transfer of substances or NPs in a living organism as well as on their potential adverse effects on the offspring. However, an extrapolation to the human situation is highly questionable since the placenta is the most species-specific organ^24,25^. Therefore, predictive *in vitro* and *ex vivo* placenta models using human cells or tissue are necessary to avoid species-specific differences. *Ex vivo* perfusion of human term placenta is considered as the “gold standard” among currently available translocation models. The use of an intact placental tissue and the establishment of a dynamic circulation is expected to yield highly *in vivo* relevant results, but a perfusion duration of only 6 hours, the low throughput and low experimental success rate are disadvantageous^26,27^. In contrast, widely used *in vitro* translocation models (i.e. a confluent monolayer of trophoblastic cancer cells cultivated on a porous membrane) are easier to handle, have a higher throughput and are more accessible for mechanistic studies. However, this model does not fully resemble the physiological structure of the placental barrier.

**Figure 1:**
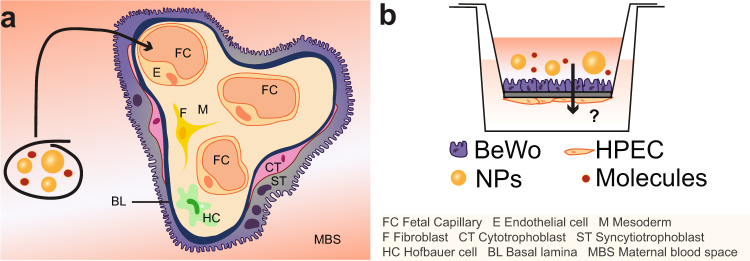
Scheme of the human placental barrier at term and the co-culture translocation model. (**a**) At the end of pregnancy, molecules or NPs present in the maternal blood stream would have to at least cross the ST layer, a basal lamina and the fetal ECs to reach the fetal circulation. (**b**) For mechanistic studies on molecule/NP translocation, a co-culture model was established consisting of a confluent layer of trophoblasts (BeWo cells) on the apical side and a monolayer of ECs (HPEC; human placental venous endothelial cells) on the basolateral side of a microporous membrane.

In the first trimester, the placental barrier is composed of four different layers, namely the syncytiotrophoblast (STB), the cytotrophoblast (CTB), the fetal stroma and the fetal endothelial cells (EC) of the capillaries (Fig. 1a). During pregnancy the placental barrier becomes thinner as the STB layer thins, fetal capillaries move to the periphery of the villi and the phenotype of the CTBs transforms from cuboidal to flat. At term, the thin network of CTBs only covers around 44% of the basal lamina surface without affecting translocation processes across the STB layer^28^. Finally, the placental tissue barrier consists of the STB, its basal lamina partly covered with a thin CTB layer and the fetal ECs, and was estimated to be around 5 µm thick^29^.

Novel *in vitro* placental models aim to improve the *in vivo* relevance by including additional relevant cell types, primary cells and/or fluidics to mimic the highly dynamic microenvironment of placental tissue. Huang *et al*. succeeded in establishing a confluent layer of human primary trophoblasts isolated from healthy term placentas on a 0.4 µm microporous membrane^30^ without the need of multiple cell seeding steps^31,32^. However, access to term placentae is restricted and isolation of primary trophoblasts is laborious and expensive. Other research groups developed first dynamic co-culture placenta models exploiting macro- or microfluidic approaches^33–35^. In all models, co-cultures of trophoblastic and endothelial cells were cultivated on either a denuded amniotic membrane^33^, a microporous PDMS (poly(-dimethylsiloxane)) membrane^34^ or a vitrified collagen membrane^35^. Glucose transport was assessed in the different models under constant flow conditions and correlated well with *ex vivo* and/or *in vivo* data. However, these models are probably only suitable for transport studies of small molecules since the chosen scaffolds/membranes likely constitute a major barrier to the free translocation of larger compounds or NPs. For instance, FITC-dextran (4.4 kDa) was highly retained by the amnion itself^33^ and small pore sizes have been shown to extensively restrict the transfer of 37 nm polystyrene NPs (PS NPs)^36^. Moreover, PDMS-based microfluidic devices are known to absorb small hydrophilic molecules and so impede reliable drug transfer studies^37–39^. To conclude, the different improvement strategies (co-cultures, primary cells, dynamic exposure) are very promising but further model optimization is urgently needed to render them suitable for translocation studies not only of small molecules but also of macromolecules and NPs.

In this study a new *in vitro* co-culture model simulating the human placental barrier was developed. To allow for translocation studies of macromolecules and NPs we chose a polycarbonate membrane with 3 µm pores as scaffold, since this type of support has been demonstrated to lead to a lower NP adherence and retardation compared to polyethylene membranes or membranes with smaller pore sizes^36^. Given that not only the syncytiotrophoblast but also the microvasculature endothelium strongly contributes to the total placental permeability^40–42^, a co-culture barrier with trophoblast cells on the apical side and endothelial cells on the basolateral side of the microporous membrane was established (Fig. 1b). Among the different trophoblast cell lines, the BeWo b30 clone best resembles human villous trophoblasts, when considering structure and function^43–46^. In addition, the relative transfer rates of small substances determined in monolayer BeWo models correlate well with transfer indices from *ex vivo* placenta perfusions^47,48^. In contrast to previous co-culture models that used macrovascular endothelial cells (e.g. HUVECs)^33–35^, we exploited a microvascular human placental venous endothelial cell line (HPEC-A2) since major phenotypic and physiologic differences exist between micro-and macrovascular endothelial cells^49–51^. To our knowledge, this is the first placental co-culture model that incorporates the two key cellular barriers of the human placenta as well as the currently most suitable porous support to assess translocation of a large variety of low to high molecular weight compounds and NPs. After a comprehensive characterization of the co-culture model, translocation studies were performed with four widely used model substances/drugs (sodium fluorescein (Na-F), FITC-dextran, indomethacin, antipyrine) as well as with 50 and 70 nm PS NPs to showcase the value of the model for mechanistic studies on NP translocation across the individual layers as well as the co-culture barrier. Translocation rates were assessed under static and shaken conditions to determine the need of a simple dynamic experimental set up, when investigating placental translocation *in vitro*, since horizontal shaking is often performed to mimic *in vivo* exposure.

## Results

### Establishment and characterization of a human placental co-culture transfer model

Here, a microporous membrane system was used to develop a co-culture model representing the human placental barrier at the end of pregnancy (Fig. 1). BeWo cells mimicking the trophoblast layer were grown on the apical side of the membrane and were co-cultured with microvascular endothelial cells (HPEC-A2) on the basolateral side. To identify a suitable medium for optimal growth and viability of the co-cultures, cells were cultivated in the recommended media for each cell type (TM or EM) as well as in a 1:1 mixture of both media for up to 5 days (see Supplementary Fig. S1). None of the different media reduced the viability of BeWo cells or HPECs. Since BeWo cells have been cultivated in a variety of different media while microvascular ECs were exclusively grown in EM, we have chosen EM as the co-culture medium.

**Figure 2:**
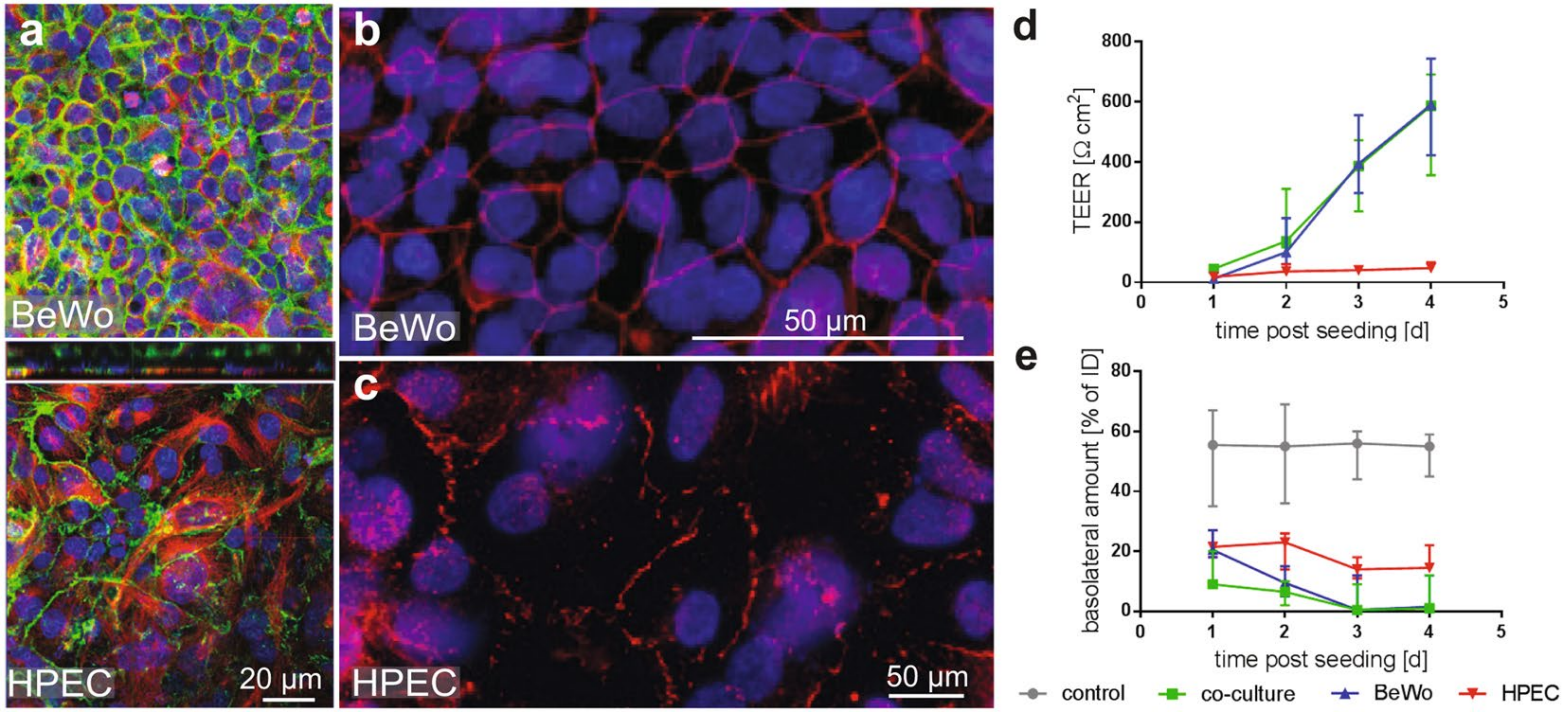
Establishment of the co-culture. (**a**) Confocal micrographs of BeWo/HPEC co-cultures after 3 d of cultivation on microporous inserts stained for γ-catenin (green, adherence junctions), tubulin (red, microtubule) and Dapi (blue, nuclei). (**b**,**c**) Images of BeWo cells (B) and HPECs (C) grown on porous membranes for 3 d and stained for ZO-1 (red, tight junctions) and Dapi (blue, nuclei). (**d**,**e**) Barrier formation was determined from day 1 to 4 after cell seeding by TEER measurements (**d**) and Na-F exclusion assays (**e**). Data represent the median ± error range (upper and lower limit) of 4 biologically independent experiments with 1 technical replicate each.

To establish a confluent monolayer BeWo cells in EM, different cell seeding numbers of 1.2 × 10^5^, 1.5 × 10^5^ and 2.0 × 10^5^ cells were applied to the membranes and formation of a tight barrier was monitored by ICC, TEER measurements and Na-F exclusion assay. Cell numbers were chosen close to the recommended seeding number of 1.0 × 10^5^ cells per cm^2^ membrane for monoculture BeWo transfer models^36,52^. Cell-cell contacts were visualized by γ- catenin staining, which indicated that a confluent cell layer was achieved after 3 d of cultivation when seeding 1.5 × 10^5^ BeWo cells (equals 1.34 × 10^5^ cells/cm^2^, Fig. 2a,b and Supplementary Fig. S2). A lower or higher cell number (1.2 × 10^5^, 2.0 × 10^5^) resulted in non-confluent cell layers or a fast overgrowth of the cells, respectively. In contrast to BeWo cells, HPECs are contact-inhibited and formed a confluent monolayer after 3 d when using an initial cell number of 1.0 × 10^5^ cells (Fig. 2a,c).

TEER values of co-cultures highly increased over time and reached 386.4 Ω cm^2^ at day 3 while Na-F translocation decreased to negligible values (basolateral amount 0.5% of ID; Fig. 2d,e). For BeWo monocultures, TEER was comparable to the co-cultures (394.5 Ω cm^2^ at d 3) whereas basal Na-F levels were slightly higher at day 1 but reached similar levels at day 3 (0.5% Na-F of ID). In contrast to BeWo cells, HPEC monocultures showed a modest increase in TEER and only a slight decrease in Na-F translocation. After 3 d of cultivation, the TEER of the HPEC monolayer reached 40.7 Ω cm^2^ and Na-F translocation was 14.0% of ID.

**Figure 3:**
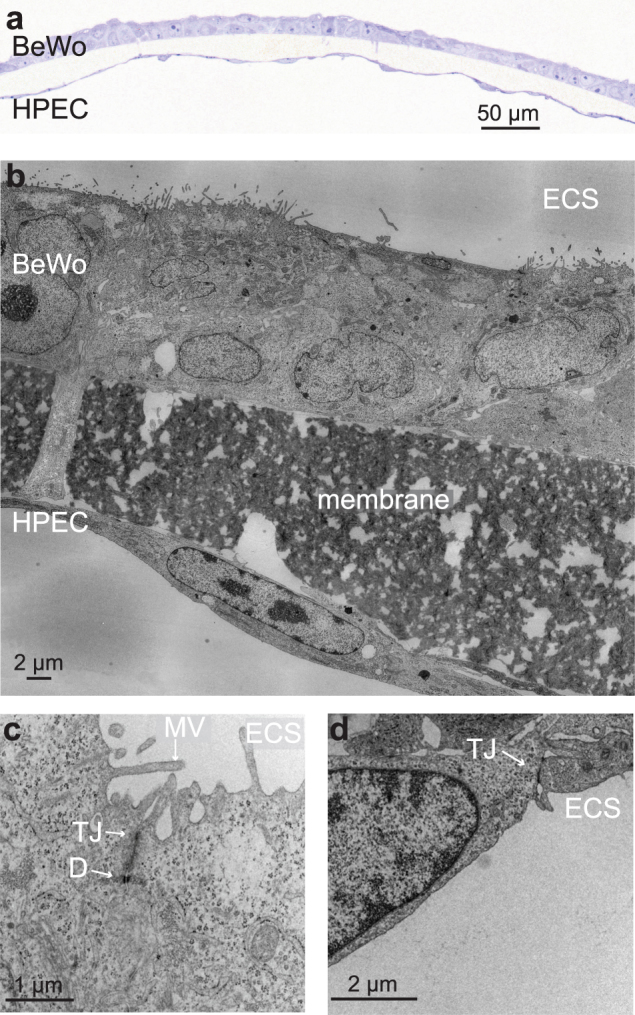
Morphological investigation of the co-culture after 3 d of cultivation. (**a**) Microscopic image of a semithin section of the co-culture (Toluidine Blue O staining). (**b**–**d**) TEM micrographs showing cellular protrusions from BeWo cells through the insert pores (**b**), microvilli on the apical side of the BeWo layer (**c**) and the formation of close cell-cell contacts by both cell types (**c**,**d**). Abbreviations: ECS, extra cellular space; D, desmosome; TJ, tight junction; MV, microvilli.

In addition, TEM and LM images of 3 d co-cultures showed that HPEC cells formed a confluent monolayer, while the BeWo cell layer was mostly present as a monolayer containing small areas where bilayer formation was evident (Fig. 3a,b). TEM micrographs further revealed the formation of tight cell-cell contacts in both cell types after 3 d of cultivation and the polarization of the BeWo layer due to microvilli formation (Fig. 3c,d).

Since the co-culture model showed placenta specific structural characteristics and formed a sufficiently tight barrier, translocation studies were conducted with different model substances and PS NPs.

### Translocation studies of model compounds and nanoparticles

Translocation studies across mono- and co-cultures were performed under static and shaken conditions. Investigation of size-dependent paracellular transport was performed using two hydrophilic substances Na-F (0.3 kDa) and FITC-dextran (40 kDa). Antipyrine, widely used as reference compound in placenta perfusion experiments, and indomethacin were used as transcellular markers, since both compounds are known to rapidly cross the human placental barrier. To investigate the applicability of this model for NP translocation studies, 49 and 70 nm PS NPs were used as model particles. NP characteristics shown in Table 1 were determined in 10% PBS solution and in EM used for translocation studies. SEM micrographs of the NPs confirmed their spherical shape and primary size (see Supplementary Fig. S3).

**Table 1:** Summary of polystyrene nanoparticle (PS NP) characteristics.

PS NP characteristics	49 nm	70 nm
Diameter (nm)^a^	49	70
Hydrodynamic diameter in 10% PBS [nm]^b^	51.7 ± 62.0	188.4 ± 108.3
Hydrodynamic diameter in EM [nm]^b^	43.3 ± 88.3	70.6 ± 109.8
Concentration in EM [particles/ml]^c^	7.73E + 12	2.65E + 11
Zeta potential in 10% PBS [mV]^b^	−44.0 ± 2.3	−25.2 ± 1.6
Zeta potential in EM [mV]^b^	−9.9 ± 1.1	−9.3 ± 0.2

^a^Data supplied by manufacturer.

^b^Experimentally determined: Hydrodynamic diameter represent mode ± SD of 5 consecutive runs. Zeta potential represent mean ± SD.

^c^Calculated concentration in the initial apical solution.

Abbreviations: EM; endothelial cell medium.

PBS; phosphate buffered saline.

To exclude that collagen-coating blocked the insert pores, transfer of Na-F, FITC-dextran and PS NPs was investigated across empty control inserts in the presence or absence of a collagen-coating (see Supplementary Fig. S4). No considerable difference between collagen-coated and uncoated membranes was observed for all substances and particles.

**Figure 4:**
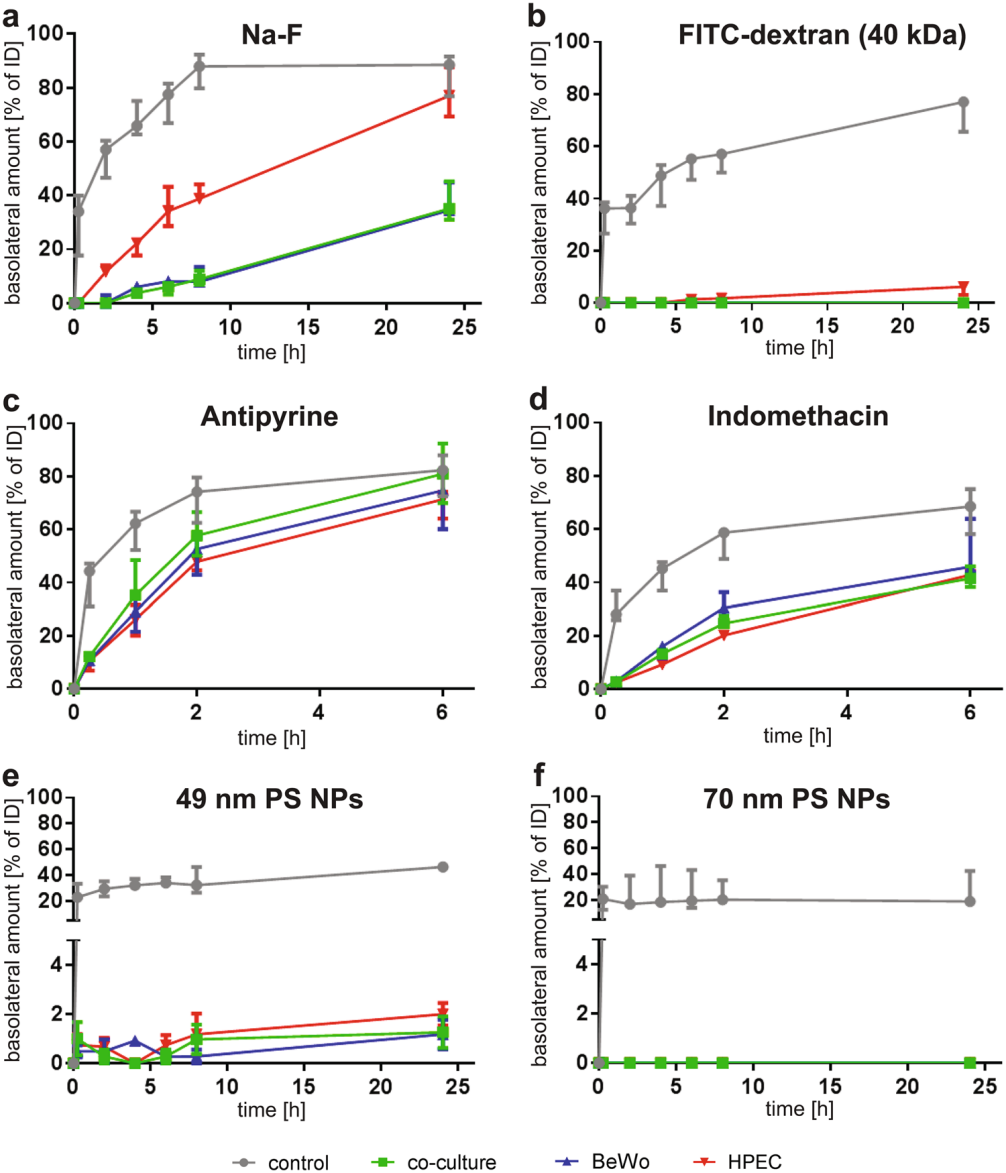
Translocation of 5 µM Na-F, 5 µM FITC-dextran (40 kDa), 100 µM antipyrine, 100 µM indomethacin, 0.5 mg/ml 49 nm PS NPs and 50 µg/ml 70 nm PS NPs across each monolayer (BeWo, HPEC) and the co-cultivated membrane under static conditions. Cells were cultivated for 3 d on collagen-coated inserts before translocation studies were performed for 24 h with Na-F (**a**), FITC-dextran (**b**), 49 nm PS NP (**e**) and 70 nm PS NP (**f**) or for 6 h with antipyrine (**c**) and indomethacin (**d**). Data represent the median ± error range (upper and lower limit) of 3–4 biologically independent experiments with 1 technical replicate each.

Transport of Na-F after 24 h across the control, BeWo, HPEC and co-culture membranes was 88.6%, 34.5%, 77.0% and 35.1% of the initial dose (ID) of Na-F, respectively (Fig. 4a). Na-F did easily pass the empty membrane. The HPEC monolayer slowed down Na-F translocation, but resulted in a similar amount after 24 h compared to the control. In contrast, both the BeWo monolayer and the co-culture considerably retained Na-F after 24 h (BeWo vs. control: p = 0.057; co-culture vs. control p = 0.029). The transfer of the larger FITC-dextran molecule (40 kDa) was blocked by all the cell layers but was still high across the control membrane (Fig. 4b). Only a small amount of 6.2% FITC-dextran, passed the HPEC layer after 24 h. Translocation of FITC-dextran was even further diminished in the presence of a BeWo monolayer or in co-cultures (median 0.0%). Translocation profiles of antipyrine and indomethacin across either BeWo and HPEC monolayers or the co-culture were highly similar (Fig. 4c,d). In contrast to indomethacin, antipyrine was transported faster and in higher amounts. After 6 h, 71.4% of antipyrine crossed the HPEC monolayer, 74.7% the BeWo layer and 80.9% the co-culture, whereas for indomethacin these values were 42.9%, 45.9% and 41.5%, respectively. When PS NPs were applied, the membrane started to contribute more substantially towards the overall barrier function but transfer across control inserts was still 46.4% for 49 nm and 18.7% for 70 nm PS NPs after 24 h (Fig. 4e,f). In the presence of the single cell layers or co-cultures, both NPs were highly retained since only 2.0%, 1.2% and 1.3% of the 49 nm PS NPs crossed the HPEC, BeWo cell layer or the co-culture after 24 h, respectively. 70 nm PS NPs were not detected on the basolateral side of any cell layer within 24 h. To assess the impact of horizontal shaking, transfer kinetics of the same substances and NPs were determined under shaken conditions. Therefore, agitation (around 50 rpm) was applied during the translocation studies to simulate a simple dynamic microenvironment. In general, similar transfer rates as in static conditions were determined for all substances and NPs (see Supplementary Fig. S5).

To compare the translocation of the different substances and NPs with each other and with published data, permeability factors were calculated according to the formulas given in the materials and methods description. First, permeability factors (P; without subtraction of the P_membrane_) were determined (equation (1); see Supplementary Table S1 for all P values) and from these apparent permeability coefficients (P_e_) across the cell layers were obtained (equation (2); Table 2). Among the different substances, antipyrine showed the highest and fastest passage across the different layers, followed by indomethacin, Na-F, 49 nm PS NPs and FITC-dextran. No translocation was detected for the 70 nm PS NPs within 24 h. Moreover, no statistical significance was found comparing the P_e_ values obtained in static and shaken conditions.

**Table 2:** Apparent permeability factors (P_e, 2h_) across the BeWo or HPEC monolayer and the co-culture.

Substance/NP	Cell layer	Static	Shaken
		P_e_ ×10^−6^ [cm s^−1^]^a^	P_e_ × 10^−6^ [cm s^−1^]^a^
Na-F	BeWo	0.0 ± 0.0	2.65 ± 0.5
	HPEC	9.0 ± 0.3	10.2 ± 0.4
	Co-culture	n.t.	1.1 ± 1.1
FITC-dextran	BeWo	n.t.	n.t.
	HPEC	n.t.	n.t.
	Co-culture	n.t.	n.t.
Antiyprine	BeWo	112.1 ± 54.1	106.4 ± 8.2
	HPEC	83.6 ± 6.5	76.5 ± 7.0
	Co-culture	400.2 ± 63.5	27.1 ± 10.7
Indomethacin	BeWo	39.5 ± 8.0	37.2 ± 11.9
	HPEC	19.0 ± 0.1	22.7 ± 0.4
	Co-culture	26.7 ± 0.2	30.7 ± 1.2
49 nm PS NPs	BeWo	0.0 ± 0.0	n.t.
	HPEC	0.5 ± 0.3	n.t.
	Co-culture	0.0 ± 0.0	0.0 ± 0.0
70 nm PS NPs	BeWo	n.t.	n.t.
	HPEC	n.t.	n.t.
	Co-culture	n.t.	n.t.

^a^Data represented as median ± mad (median absolute deviation) of 3–4 biologically independent experiments with 1 technical replicate each (n.t.: no translocation detected).

## Discussion

Translocation studies at the placental barrier are key to predict potential fetal exposure and teratogenic effects. A comprehensive knowledge on uptake and translocation mechanisms is indispensable for the safe design and use of drugs and NPs in industrial and medical applications. Predictive human placenta models are a prerequisite to achieve meaningful results since the placenta is considered to be the most species-specific mammalian organ. Although complex *ex vivo* placenta models exist, their applicability for mechanistic translocation studies is limited due to the restricted access to human placental tissue or high donor-to-donor variations, among others. Consequently, there is a strong need for advanced *in vitro* alternatives but the most frequently used BeWo transfer model does not take into account the multilayered placental barrier structure or the highly dynamic environment of the physiological situation. First studies highlighting the importance of including endothelial cells in placental translocation models are emerging^33,53^. For instance, it has been shown that the endothelial cell layer seems more resistive to glucose transfer than the trophoblast layer^54^.

Therefore the aim of this study was to increase the predictive value of the widely used *in vitro* BeWo transfer model and to make it suitable for transfer studies of large molecules and NPs. Co-cultures of trophoblast cells and microvascular endothelial cells were established on microporous membranes with 3 µm pore sizes. Larger pore sizes would lead to transmigration of the cells through the membrane pores while smaller pore sizes are imposing a major barrier for the free transfer of larger compounds or NPs. After optimization of the cultivation conditions, tight cell layers were obtained already 3 days after cell seeding. In contrast, published BeWo transfer models mostly require 5–6 days to achieve confluency. The reason for the faster BeWo monolayer formation was most likely due to a combination of a slightly increased cell seeding number and the use of a different cultivation media. Importantly, the increased seeding number did not result in multiple cell layer formation. Polarization of the trophoblast layer was verified by the presence of microvilli at the apical surface, and is an important key characteristic that determines specific asymmetric transport of e.g. iron^55^. Na-F exclusion and TEER measurements confirmed the formation of a tight barrier suitable for translocation studies across the individual monolayers and the co-culture.

To verify our model with previous *in vitro*, *ex vivo* and *in vivo* findings we conducted translocation studies with the model substances Na-F, FITC-dextran (40 kDa), antipyrine and indomethacin, and additionally with two different sized PS NPs under static and shaken conditions. Compounds <500 Da are known to cross the human placental barrier easily, whereas bigger compounds passing poorly or not at all^56^. To confirm a size-dependent transport, we investigated the translocation of Na-F (0.3 kDa) and FITC dextran (40 kDa), previously used as markers for paracellular passive diffusion^54^. Recently Na-F was also found to be a substrate of organic anion transporting polypeptides (OATP), which are present in BeWo cells and placental tissue *in vivo*^57–60^. As expected for an intact placental barrier, substantial amounts of Na-F passed the placental barrier in our co-culture model after 24 h whereas the high-molecular weight FITC-dextran (40 kDa) was almost completely retained in the apical chamber. Our transport studies on the individual monolayers further revealed a higher translocation of both markers across the HPEC layer than the BeWo layer, suggesting that the trophoblast barrier is tighter than the endothelial barrier. Moreover, the barrier capacity of the co-culture was equal to the one of the BeWo layer, which indicates that the BeWo cells constitute the key cell barrier layer of the placenta for paracellular transport.

Antipyrine is widely used as a reference compound in *ex vivo* perfusions and *in vitro* translocation studies and exhibits a fast translocation across the placental barrier via transcellular passive diffusion^5,26,47,48,61–67^. Indomethacin is known to easily pass the human placenta *in vitro*^47^, *ex vivo*^68^ and *in vivo* throughout gestation^69,70^. In our newly established co-culture model, a fast transport of indomethacin and antipyrine across the trophoblast and endothelial layer within 24 h was confirmed. In a previous study using the BeWo monoculture transfer model, Li *et al*. reported P_1h_ values of 38.0 × 10^−6^ cm s^−1^ and 23.2 × 10^−6^ cm s^−1^ for antipyrine and indomethacin transfer under static conditions, respectively^47^. Other studies reported permeability values for antipyrine crossing a BeWo monolayer of P_1h_ of 53 × 10^−6^ cm s^−1^ under static conditions^67^ and P_e_,_1h_ of 58 × 10^−6^ cm s^−1^ under shaken conditions^48^. These values were highly similar to the permeability values measured in this study for antipyrine (P_1h_: 36.2 × 10^−6^ cm s^−1^ static; P_e, 1h_: 79.3 × 10^−6^ cm s^−1^ shaken) and indomethacin (P_1h_: 19.9 × 10^−6^ cm s^−1^ static). Importantly, it has been shown that the relative transfer rates of small substances determined in monolayer BeWo models correlate well with transfer indices from *ex vivo* placenta perfusions^47,48,71^, suggesting that our model delivers results with a high predictive value.

The applicability of our co-culture model for NP translocation studies was evaluated using PS NPs since for those NPs, there is a reasonable amount of *in vitro* and *ex vivo* data available for comparison. 70 nm PS NPs did not cross the *in vitro* placental barrier at all (mono- and co-culture), whereas small amounts of 49 nm PS NPs (1–2% of ID after 24 h) were detected in the basolateral compartment. A similar limited transport of 3.5% of 50 nm PS NPs across BeWo monolayer cultures has been previously reported^36^. In addition, a size-dependent transfer of PS NPs has been shown in recent *ex vivo* and *in vitro* placental transfer studies^36,64^.

Previous studies often determined placental transfer across the *in vitro* barrier under simple dynamic conditions (constant stirring or shaking) to mimic *in vivo* blood flow in placental tissue^17,18,36,48,63,67,72^. This is of particular relevance for NPs, which exhibit unique sedimentation/agglomeration behavior and transport kinetics in biological media^73,74^. Dynamic exposure has been shown to reproduce a more predictable dose compared to static conditions^75^. However, in the BeWo transfer model, the impact of horizontal shaking during translocation studies compared to static conditions has never been verified. Here, we assessed the influence of a constant horizontal shaking on placental translocation of a broad variety of substances ranging from low molecular weight compounds to comparably large NPs and covering both para- and transcellular routes. Translocation profiles were highly similar in static and shaken conditions for all substances and NPs. Small trends of an increased antipyrine and indomethacin translocation under shaken conditions were seen compared to static conditions after 24 h, but no significance was found. Since the permeability values were very similar and the transfer ranking of the compounds was the same in static and shaken conditions, we suggest that horizontal shaking is not sufficient to mimic physiological maternal and fetal blood flow. Consequently, future studies should explore if a truly dynamic model using microfluidic or macrofluidic approaches as well as the establishment of the barrier cultures under constant flow is more effective in improving the predictive value of *in vitro* placental transfer models. First studies in this direction revealed that cell morphology and glucose transport was more *in vivo* relevant in placenta-on-a-chip devices with integrated liquid flow^34,35^. Also for NPs, chip-based dynamic cell cultures reproducing physiological relevant shear stress conditions enabled more accurate NP uptake studies^76^. However, despite the fact that these novel placental *in vitro* models apparently improve physiological parameters by application of trophoblast-endothelial co-cultures and physiological flow conditions, unfavorable properties of the chip material or the membrane scaffolds often limit the applicability of these models for translocation studies tremendously^37–39^. Therefore, the traditional BeWo transfer model still seems to be the most appropriate placental translocation system, which allows ranking of transfer rates of small compounds in agreement with *ex vivo* and *in vivo* outcomes.

To conclude, we successfully developed a novel placental co-culture model suitable not only for translocation studies of small compounds, but also of macromolecules and even NPs. The application of different cell layers enables mechanistic investigations of their specific impact on translocation and permeability. The applied modifications render our multicellular model more versatile, reliable, cost- and time-effective and thereby potentially more attractive for the pharmaceutical industry. In the future, inclusion of additional cell types (e.g. immune cells) and incorporation of the placental co-culture model into a perfused system may allow to further reduce the gap between *in vitro* and *in vivo* studies, thus helping to refine, reduce and replace experimentation in pregnant animals.

## Materials and Methods

### Cell culture

HPEC-A2 cells (SV40-transformed microvascular human placental venous endothelial cells) were obtained from Prof. G. Desoye (Department of Obstetrics and Gynecology, Medical University Graz, Graz, Austria) with permission from Prof. P. Friedl (Institute of Biochemistry, Technical University Darmstadt, Darmstadt, Germany) and cultivated in endothelial cell growth medium MV supplemented with 1 vial SupplementMix according to the manufacturer’s guide (PromoCell, Heidelberg, Germany) and 1% penicillin/streptomycin (pen/strep, Gibco, Luzern, Switzerland), which will be referred to as endothelial cell medium (EM). The human placental choriocarcinoma cell line BeWo b30 was kindly provided by Prof. Dr. Ursula Graf-Hausner (Zurich University of Applied Science) with permission from Dr. Alan L. Schwartz (Washington University School of Medicine, MO, USA). BeWo cells were propagated in trophoblast medium (TM), which is Ham’s F-12K medium supplemented with 10% fetal calf serum (FCS, Invitrogen, Basel, Switzerland), 1% pen/strep (Gibco, Luzern, Switzerland) and 2 mM L-Glutamine (Gibco, Luzern, Switzerland). Both cell lines were sub-cultured twice a week using trypsin-EDTA solution and cultivated in a humidified incubator at 37 °C with 5% CO_2_ atmosphere.

### Monolayer and co-culture formation on microporous inserts

**Figure 5:**
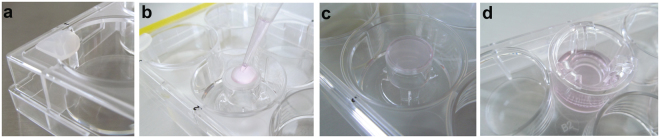
Seeding of the cells on the basolateral side of the insert. (**a**) Small rubber spacers are placed in each corner of a 6-well plate to avoid direct contact between membrane and lid. (**b**) 1 ml PBS is added to one well to humidify the surrounding. The reversed insert is placed into this well and 1 × 10^5^ HPECs in 200 µl are added to the membrane. (**c**) The lid is closed immediately. Adhesion of the drop prevents cell aggregation in the center and medium loss. (**d**) The insert is placed back into a 12-well plate after 2 h incubation at 37 °C/5% CO_2_.

Polycarbonate Transwell^®^ inserts (pore size 3.0 µm, growth area 1.12 cm^2^, apical volume 0.5 ml, basolateral volume 1.5 ml; Corning^®^, Sigma-Aldrich, Buchs, Switzerland) were pre-coated with 50 µg/ml human placental collagen IV (Sigma-Aldrich, Buchs, Switzerland) for 1 h at 37 °C/5% CO_2_ (200 µl on both sides). Then the membrane was washed twice with phosphate buffered saline (PBS, pH 7.4, Sigma-Aldrich, Steinheim, Germany). Translocation experiments were done either with BeWo or HPEC monocultures or with both cell lines co-cultivated on a membrane. For the BeWo monolayer 1.5 × 10^5^ cells in 500 µl EM were added apically and 1.5 ml EM basolaterally. For the HPEC monolayer the cells were seeded on the basolateral side of the membrane (Fig. 5a–d). Therefore, the reversed insert was placed in a 6-well plate (TPP, Faust AG, Schaffhausen, Switzerland), which had been filled with 1 ml sterile PBS to ensure a humid surrounding. Small rubber spaces (1.5 mm thick) were placed in each corner of the plate in order to slightly lift the lid to prevent direct contact of the lid with the membrane. This enables the adhesion of the drop to the lid, thereby avoiding medium loss as well as cell aggregation in the center. 1 × 10^5^ HPECs in 200 µl EM were added to the membrane and the lid was immediately placed on the plate. After 2 h of incubation (37 °C, 5% CO_2_), the insert was placed into a 12-well plate with fresh EM (0.5 ml apical and 1.5 ml basolateral). For the co-culture, the steps described above for the monocultures were done, starting with the HPEC layer. The inserts were cultivated for 3 d at 37 °C/5% CO_2_ in EM (medium change after 48 h) under static conditions.

### Transepithelial electrical resistance (TEER)

Barrier formation was evaluated by TEER measurement using a chopstick electrode (STX3, World Precision Instruments Inc., Sarasota, USA). TEER was determined on the collagen-coated inserts in the presence or absence of cells. TEER values for the cell layer were obtained by subtracting the intrinsic resistance (blank insert membrane) from the total resistance (insert membrane with cells) and were corrected for the surface area (Ω cm^2^).

### Sodium fluorescein (Na-F) exclusion assay

To determine barrier formation, exclusion of Na-F was determined daily for 4 d. 0.5 ml of 5 µM Na-F (Sigma-Aldrich, Buchs, Switzerland) was added to the apical chamber and 1.5 ml EM (without phenol red) to the basal chamber. After incubation for 3 h at 37 °C/5% CO_2_, Na-F was detected in basolateral samples (50 µl) using a microplate reader (Mithras^2^ LB 943, Berthold Technologies GmbH, Zug, Switzerland; excitation 485 nm, emission 528 nm).

### Immunocytochemistry (ICC)

For ICC stainings inserts were fixed in 4% paraformaldehyde (PFA; Sigma-Aldrich, Buchs, Switzerland)/0.2% Triton X-100 (Sigma-Aldrich, Buchs, Switzerland) for 10 min at RT. Afterwards inserts were blocked in 5% goat serum (in PBS) at RT for 1 h. Primary rat-anti-tubulin (Abcam, Cambridge, UK; 1:1000) and mouse-anti-(γ)-catenin (BD biosciences, Allschwil, Switzerland; 1:500) or mouse-anti-ZO-1 (Invitrogen,Frederick, MD, USA; 1:100) diluted in 0.5% BSA (Sigma-Aldrich, Buchs, Switzerland) in PBS were applied for 60 min at room temperature (RT; tubulin, γ-catenin) or overnight at 4 °C (ZO-1), followed by incubation with Alexa Fluor A488 goat anti-mouse (lifetechnologies, Eugene, OR, USA; 1:400), Alexa Fluor A555 goat anti-rat (Invitrogen, Eugene, OR, USA; 1:400) or Alexa Fluor A546 goat anti-mouse (Invitrogen, Eugene, OR, USA; 1:400) diluted in 0.5% BSA in PBS for 60 min at RT. 40,6-diamidin-2-phenylindol (DAPI; Sigma-Aldrich, Buchs, Switzerland; 10 min at RT, 1:1000) was included during one PBS washing step. Control images were taken from cells treated with the different secondary antibodies only to demonstrate antibody specificity (see Supplementary Fig. S6).

To obtain flat membranes, whole inserts were embedded with Mowiol 4-88 (Sigma-Aldrich, Buchs, Switzerland) and membranes were cut off from the holder with a scalpel after drying of the Mowiol 4-88. Images were acquired with an Axio Imager 2 (Zeiss, Feldbach, Switzerland) or a confocal laser scanning microscope (CLSM 780, Zeiss, Feldbach, Switzerland). Z-stack images were obtained with the CLSM.

### Semithin and ultrathin sections

Transmission electron microscopy (TEM) was conducted to investigate the morphological details. Inserts were fixed in 3% glutaraldehyde in 0.1 M sodium cacodylate buffer and were washed in 0.2 M sodium cacodylate buffer. After a post-fixation step in 2% osmium tetroxide in 0.1 M sodium cacodylate buffer, samples were dehydrated through a graded ethanol series followed by acetone and finally embedded in Epon resin (Sigma-Aldrich, Buchs, Switzerland). Ultrathin sections were contrasted with 2% uranyl acetate and lead citrate (Reynolds 1963) before imaged in a Zeiss EM 900 (Carl Zeiss Microscopy GmbH, Germany) at 80 kV. To visualize barrier thickness transversal sections (450 nm) were stained with Toluidine Blue O and imaged by light microscopy (Leica DM4000 B LED, Leica Microsystems CMS GmbH, Wetzlar, Germany).

### Nanoparticle dispersion and characterization

Plain, green-fluorescent polystyrene NPs (PS NPs) of 49 and 70 nm were commercially available from Kisker GbR (Steinfurt, Germany and Spherotech (Lucerne, Switzerland). Stock solutions were vortexed before each use, diluted to the required concentration in EM and immediately added to the cells. The hydrodynamic diameter was measured in 10 µg/ml (49 nm PS NPs in EM, 70 nm PS NPs in EM or 10% PBS) or 100 µg/ml NP solutions (49 nm PS NPs in 10% PBS) using Nanoparticle Tracking Analysis (NTA 3.1 Build 3.1.54; Nanosight NS500, Malvern, Worcestershire, UK). The concentration of the particles in the maternal solution was calculated considering a density for PS of 1.05 g cm^−3^. The zeta potential of the NPs was measured from 10 µg/ml dilutions in EM and 10% PBS (Zetasizer Nanoseries, Nano-ZS90, Malvern, Worcestershire, UK).

### Translocation studies

In order to investigate placental transport without adversely affecting the integrity of the cell layers, the toxic potential of the NPs was investigated using the MTS assay (see Supplementary Fig. S7). Concentrations of up to 100 µg/ml of 70 nm PS NPs did not decrease the viability of BeWo cell or HPECs after 24 h of treatment. Previous publications showed that also 49 nm PS NPs, antipyrine, indomethacin, Na-F and FITC-dextran (40 kDa) did not affect the viability of BeWo cells^47,64^. Therefore reported concentrations were chosen for translocation studies of the model compounds and NPs to allow a comparison to previous results. These were 5 µM Na-F, 5 µM FITC-dextran (40 kDa), 100 µM antipyrine, 100 µM indomethacin, 0.5 mg/ml 49 nm PS NPs and 50 µg/ml 70 nm PS NPs.

Translocation studies were performed with co-cultured membranes as well as separate monolayers (BeWo on the apical or HPEC on the basolateral side of the insert) cultivated for 3 d as described above. Transfer studies with Na-F, FITC-dextran (40 kDa; Sigma-Aldrich, Buchs, Switzerland) and PS NPs were conducted using phenol red-free EM to avoid interference with fluorescence spectroscopic measurements. Na-F, FITC-dextran, 70 nm PS NPs and 49 nm PS NPs were added to the apical chamber. At each time point (0, 0.25, 2, 4, 6, 8, 24 h) samples (50 µl) were taken from the basolateral chamber and renewed with fresh medium. Fluorescent signals of the samples were measured at an excitation of 485 nm and an emission of 528 nm using a microplate reader (Mithras^2^ LB 943, Berthold Technologies GmbH, Zug, Switzerland). Translocation of antipyrine (Sigma-Aldrich, Buchs, Switzerland) and indomethacin (Sigma-Aldrich, Buchs, Switzerland) was investigated applying the same experimental conditions. Only differences were the use of EM with phenol red and sample volumes of 200 µl (basolateral) at each time point (0, 0.25, 1, 2, 6 h). Analysis of these samples was done by high performance liquid chromatography (HPLC). These studies were conducted in static and shaken (horizontal shaking at 50 rpm) conditions to determine the potential influence of simple shaking on transfer rates.

The mass transported (ΔQ_n_) was calculated for each time point and corrected for the mass taken before:

1 $${\rm{\Delta }}Qn={C}_{n}\ast {V}_{w}+\,\sum _{j=1}^{n-1}{V}_{s}\ast {C}_{j}$$

with the concentration measured at time t_n_ (C_n_), the volume of the well (V_w_, 1.5 ml) and the sample volume (V_s_). The sum of the amount removed during previous sampling is added respectively (Σ). Results were then expressed as basolateral amount of the initial dose (ID) in %. Equilibrium between the apical and basolateral chamber would be reached if 75% of the ID would pass the barrier and would be found in the basolateral chamber (V_apical_ 0.5 ml, V_basolateral_ 1.5 ml). The permeability factor was calculated with the following two equations:

2 $$P=\frac{{\rm{\Delta }}Q/{\rm{\Delta }}t}{A\ast {C}_{0}}$$

3 $${P}_{e}=\frac{1}{(\frac{1}{{P}_{c}}-\frac{1}{{P}_{m}})}$$

where the permeability factor (P; cm s^−1^) is first calculated as the quotient of the amount transported (ΔQ; mg) at a specific time point (Δt; sec) divided by the product of the membrane surface area (cm^2^) and the initial concentration of the substance (C_0;_ mg cm^−3^). P_e_ describes the apparent permeability factor corrected for the influence of the membrane. Therefore, the permeability value across the membrane P_m_ was subtracted from the permeability across the cells (P_c_; monolayer or co-culture).

### High performance liquid chromatography (HPLC)

Antipyrine was detected using a HP Series 1200 high-performance liquid chromatograph (Agilent Technologies, Waldbronn, Germany) equipped with a UV detector. 40 µl of each sample were applied to a Poroshell 120 EC-C-18 column (2.7 µm, 100 × 2.1 mm; Agilent Technologies AG, Basel, Switzerland). For analysis of indomethacin the same sample volume was injected on an EC 100/2 Nucleodur 100-3 C18ec column (3.0 µm, 100 × 2 mm; Macherey Nagel AG, Oensingen, Switzerland). The detector was set at 245 nm and 270 nm to investigate the presence of antipyrine and indomethacin, respectively.

For antipyrine measurements, the initial mobile phase consisted of 95% eluent A (0.07 M KH_2_PO_4_ in Milli-Q water, pH 3.5; Sigma-Aldrich, Buchs, Switzerland) and 5% eluent B (methanol plus 0.04% formic acid, pH 3.5; Sigmal-Aldrich, Buchs, Switzerland & Fisher Scientific, Loughborough, UK, respectively), which then increased linearly to 60% after 12 min, where it stayed for 8 min. Then the proportion of eluent B decreased back to 5% within 2 min, where it equilibrated for 13 min until the next measurement. The flow rate was set to 0.13 ml/min during the analysis. Indomethacin was measured using 0.2% formic acid in nanopure water (A; Barnstead Nanopure, Thermo Fisher Scientific, Switzerland) and 0.2% formic acid in acetonitrile (B; Acros Organics, Fisher Scientific AG, Wohlen, Switzerland). The proportion of eluent B was constant for the first 3 min at a flow rate of 0.250 ml/min, then increased linearly during 5 min to 60%, where it stayed constant for 10 min. Within 2 min the amount of eluent B decreased to 15% and was then constant for 10 min.

### Statistical analysis

Data are represented as median ± error range (upper and lower limit) or median ± median absolute deviation (mad) from 3 independent biological experiments with 1 technical replicate each unless stated otherwise. A non-parametric two-tailed Mann Whitney test was performed to find statistical significance between translocation of each compound/NP across the different cell layers compared to their respective control (% of ID) or between static and shaken conditions (P_e_ values). A p-value < 0.05 was considered significant and respective comparisons and calculated p-values are mentioned in the text. The analysis was done using GraphPad Prism (GraphPad Prism version 6, GraphPad Software, La Jolla California USA, http://www.graphpad.com).

### Data availability

The datasets generated during and/or analysed during the current study are available from the corresponding author on reasonable request.

## Electronic supplementary material

Supplementary Information
