# Supplementary material for: An advanced human in vitro co-culture model for translocation studies across the placental barrier
Source: Sci Rep. 2018 Mar 29;8:5388. doi: 10.1038/s41598-018-23410-6 (PMC5876397; doi:10.1038/s41598-018-23410-6)
Supplement: Supplementary file 1 — Supplementary Information [file 41598_2018_23410_MOESM1_ESM.pdf]

Supplementary data

Scientific Reports

An advanced human *in vitro* co-culture model for translocation studies across the placental barrier

Leonie Aengenheister[1], Kerda Keevend[1], Carina Muoth[1], René Schönenberger[2], Liliane Diener[1], Peter Wick[1], Tina Buerki-Thurnherr[1,*]

[1] Particles-Biology Interactions, Empa, Swiss Federal Laboratories for Materials Science and Technology, Lerchenfeldstrasse 5, 9014 St. Gallen, Switzerland

[2] UTOX, EAWAG, Swiss Federal Institute of Aquatic Science and Technology, Ueberlandstrasse 133, 8600 Dübendorf, Switzerland

*tina.buerki@empa.ch

Materials and Methods

Characterization of PS NPs by scanning electron microscopy (SEM)

SEM was used for morphological studies. Small drops of each NP stock solution were applied on a carbon film attached to glass slips and left to dry at RT for 1 d (light protected). Before SEM imaging (Hitachi S-4800, Japan), samples were sputter-coated with a layer of palladium/gold alloy (5 nm thick; Au/Pd: 80/20).

Determination of BeWo cell seeding number

To determine the optimal BeWo cell seeding number, different cell numbers (1.2×10^5, 1.5×10^5, 2.0×10^5) were seeded on the membranes, and formation of a confluent layer was followed over the course of 4 consecutive days. The cells were incubated under static conditions at 37°C/ 5% CO_2 in EM (medium change after 48 h). The γ-catenin staining was done as indicated in the methods. Overview of the complete membrane was constructed from 318 individual tiles acquired with an Axio Imager 2 (Zeiss, Feldbach, Switzerland), which were automatically stitched to one overview image.

MTS viability assay

The influence of different media on cell viability as well as on the *in vitro* cytotoxicity of 70 nm PS NPs and Ag NPs was assessed using the MTS viability assay. BeWo and HPEC-A2 cells were seeded in a 96-well plate (8000 cells per well for influence of different media; 10000 cells per well for effect of NPs; 3 replicates were done per condition and experiment) for 24 h and subsequently treated with different media or different concentrations of PS NPs. As negative control, cells without treatment were used and as positive control 1 mM $CdSO_4$ was applied. After incubation (3 or 5 d for medium test; 24 h for

NPs) at 37°C and 5% CO_2, the MTS assay (CellTiter96® AQueous One Solution Cell Proliferation Assay; Promega) was performed according to the manufacturer's instructions. Optical density was measured at 490 nm with a microplate reader (Mithras^2 LB 943, Berthold Technologies GmbH, Zug, Switzerland). OD values were blank-corrected and normalized to untreated controls or optimal cell culture medium.

Influence of collagen coating on substance/ NP adhesion

Potential stronger adherence to collagen-coated membranes compared to untreated membranes was determined for Na-F, FITC-dextran 40 kDa and 70 nm PS NPs. Therefore, either 200 µl of 50 µg/ml human placental collagen or 200 µl of PBS (- collagen) were added to each side of the membrane. After incubation for 1 h at 37°C/ 5% CO_2, the inserts were washed twice with PBS. According to the experimental protocol of the translocation studies, 5 µM Na-F, 5 µM FITC-dextran 40 kDa or 50 µg/ml 70 nm PS NPs were added in 500 µl EM (without phenol red) to the apical chamber. Inserts were incubated for 24 h (37°C/ 5% CO_2, static) and 50 µl samples were taken after 0, 0.25, 2, 4.5, 6, 8 and 24 h from the basolateral chamber (total volume 1.5 ml, 50 µl fresh medium was added after each sampling). Fluorescence was measured with a microplate reader (Mithras^2 LB 943, Berthold Technologies GmbH, Zug, Switzerland; excitation 485 nm, emission 528 nm). The mass transported was calculated for each time point and corrected for the mass removed previously.

Results

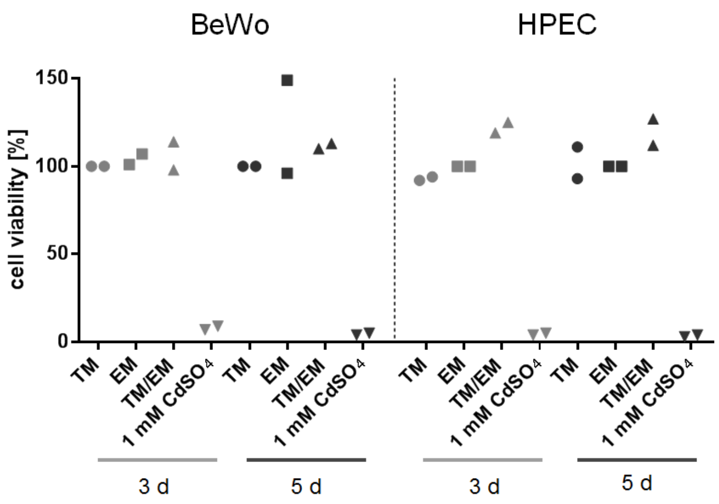

Fig. S1 Influence of different media on cell viability. BeWo cells and HPECs were cultivated in TM, EM or a mixture of these media (1:1) for 3 and 5 d before cell viability was determined by MTS. BeWo cells were routinely cultivated in TM and HPEC in EM and cell viability in these conditions were set to 100%. 1 mM $CdSO_4$ is used as positive control. Data represent the mean from 3 technical replicates of two biologically independent experiments.

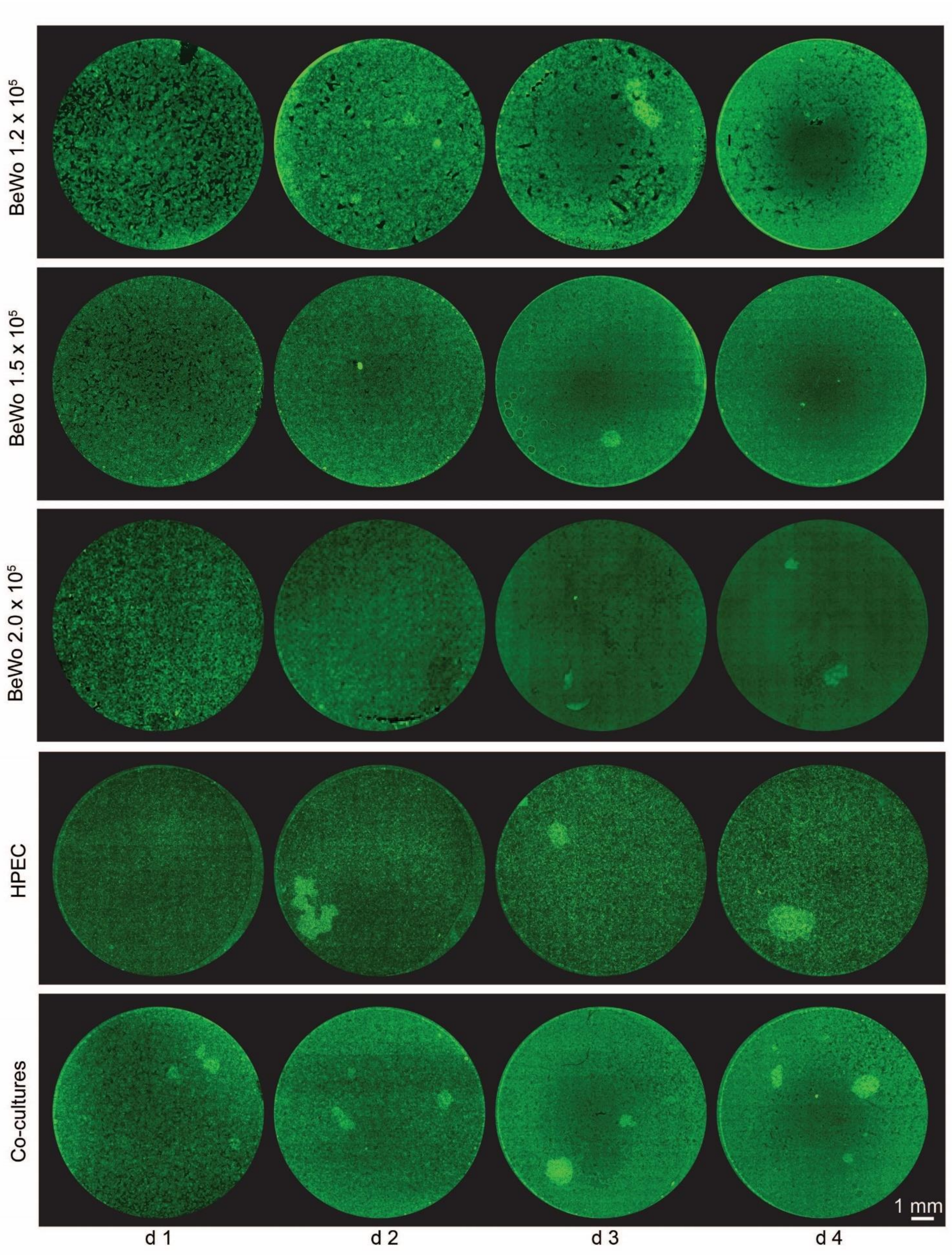

Fig. S2 Establishment of the cell seeding number. γ-catenin staining of the whole insert after 1-4 d of cultivation under static conditions. A confluent cell layer was achieved after 3 d of cultivation when seeding 1.5 x 10^5 BeWo cells. Lower or higher cell seeding numbers (1.2 x 10^5, 2.0 x 10^5) resulted in non-confluent cell layers (black spots represent cell-free areas) or a fast overgrowth of the cells, respectively.

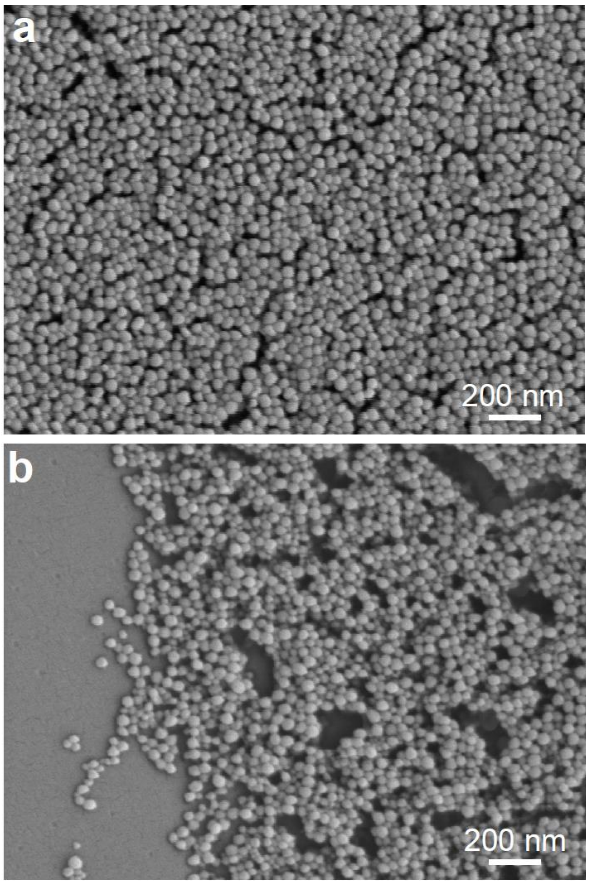

Fig. S3 SEM micrographs of PS NPs. **a** 49 nm PS NPs; **b** 70 nm PS NPs.

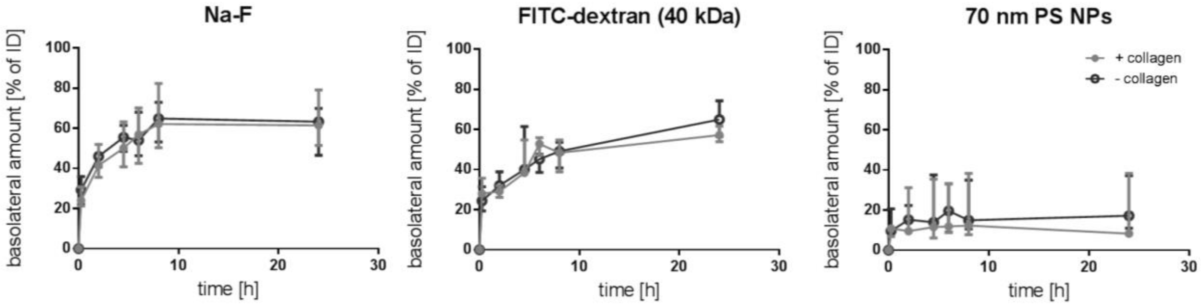

Fig. S4 Influence of collagen-coating on the translocation of Na-F, FITC-dextran (40 kDa) and 70 nm PS. Transfer was determined across empty control inserts in the presence or absence of a collagen-coating. Data represent the median ± error range (upper and lower limit) of 3-4 biologically independent experiments with 1 technical replicate each.

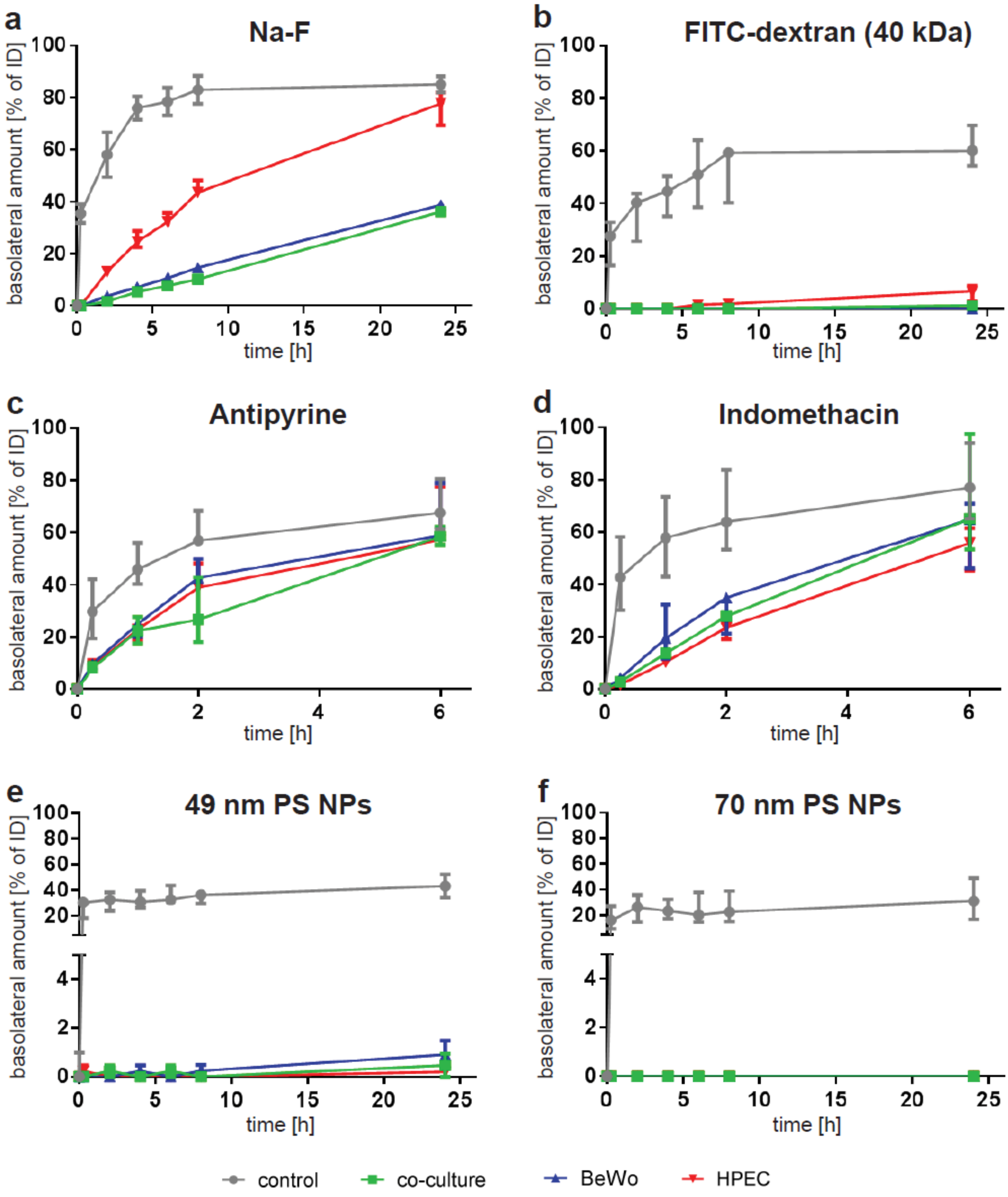

Fig. S5 Translocation of 5 µM Na-F, 5 µM FITC-dextran (40 kDa), 100 µM antipyrine, 100 µM indomethacin, 0.5 mg/ml 49 nm PS NPs and 50 µg/ml 70 nm PS NPs across each monolayer (BeWo, HPEC) and the co-cultivated membrane under shaken conditions. Cells were cultivated on collagen-coated inserts for 3 d before translocation experiments were performed for 24 h with Na-F (a), FITC-dextran (b), 49 nm PS NP (e) and 70 nm PS NP (f) or for 6 h with antipyrine (c) and indomethacin (d). Data represent the median ± error range (upper and lower limit) of 3-4 biologically independent experiments with 1 technical replicate each.

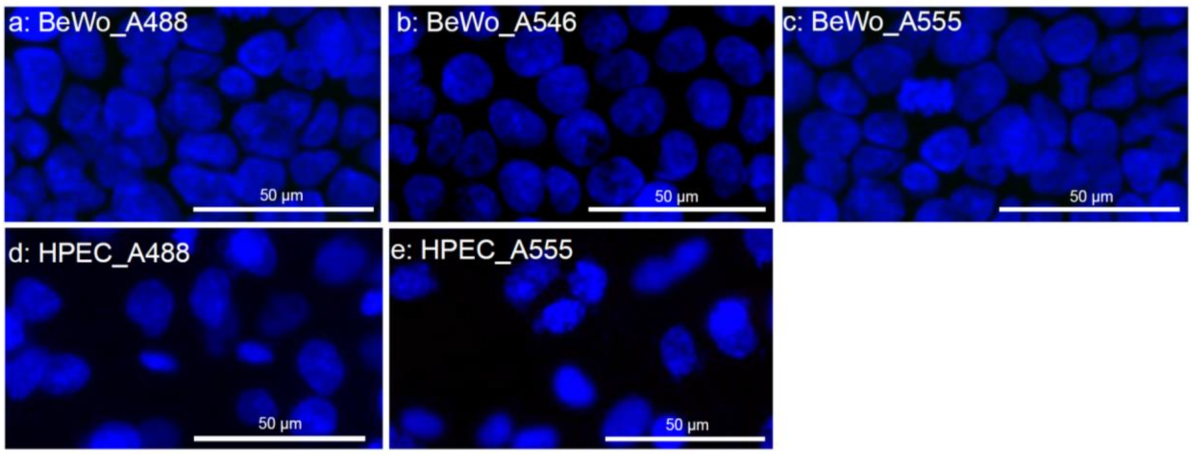

Fig. S6 Controls for the immunostaining of ZO-1, catenin and tubulin. To demonstrate antibody specificity BeWo and HPEC cells were cultivated on the membranes as described in the manuscript and stained with the different secondary antibodies only. BeWo and HPEC cells were treated with Alexa Fluor A488 goat anti-mouse (a, d), Alexa Fluor A546 goat anti-mouse (b) or Alexa Fluor A555 goat anti-rat (c, e) antibodies only, which were used for catenin, ZO-1 and tubulin staining, respectively.

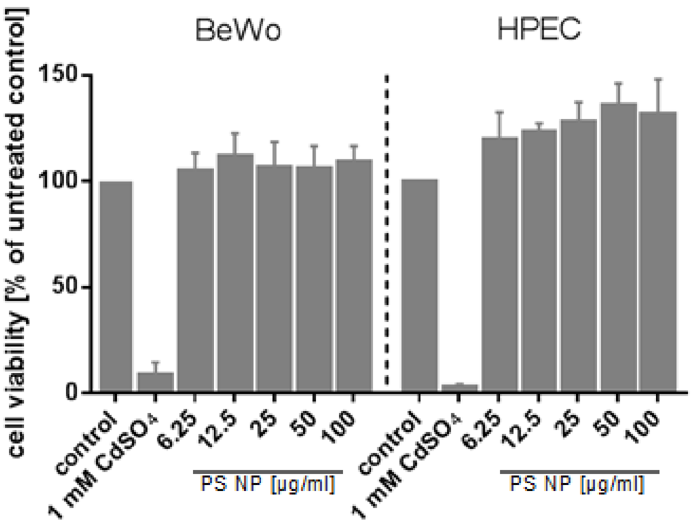

Fig. S7 Effect of 70 nm PS NP on BeWo and HPEC viability. Cell viability of BeWo and HPEC after 24 h of treatment with 6.25, 12.5, 25, 50, 100 µg/ml 70 nm PS NPs was determined by an MTS assay. 1 mM $CdSO_4$ is used as positive control. Data represent the mean ± s.e.m. of three biologically independent experiments with 3 technical replicates each.

Table S1: Permeability factors (P_{2h}) across the control membrane, BeWo or HPEC monolayer and the co-culture

Substance/NP	Cell layer	static<br>P x 10^{-6} [cm s^{-1}][a]	shaken<br>P x 10^{-6} [cm s^{-1}][a]
Na-F	Control	35.3 ± 1.5	37.6 ± 5.5
	BeWo	0.0 ± 0.0	2.5 ± 0.4
	HPEC	7.2 ± 0.26	8.1 ± 0.2
	Co-culture	n.t.	1.0 ± 1.0
FITC-dextran	Control	22.6 ± 2.9	25.0 ± 2.1
	BeWo	n.t.	n.t.
	HPEC	n.t.	n.t.
	Co-culture	n.t.	n.t.
Antiyprine	Control	50.0 ± 3.4	35.3 ± 0.7
	BeWo	32.6 ± 2.8	26.5 ± 1.1
	HPEC	29.7 ± 0.4	24.1 ± 1.0
	Co-culture	35.8 ± 4.7	16.5 ± 5.3
Indomethacin	Control	36.4 ± 1.1	39.6 ± 6.5
	BeWo	19.0 ± 3.5	21.7 ± 0.3
	HPEC	12.6 ± 0.6	14.6 ± 1.2
	Co-culture	15.3 ± 1.1	17.3 ± 0.8
49 nm PS NPs	Control	18.3 ± 3.5	20.3 ± 3.5
	BeWo	0.0 ± 0.0	n.t.
	HPEC	0.5 ± 0.3	n.t.
	Co-culture	0.0 ± 0.0	0.0 ± 0.0
70 nm PS NPs	Control	9.9 ± 2.9	16.4 ± 5.8
	BeWo	n.t.	n.t.
	HPEC	n.t.	n.t.
	Co-culture	n.t.	n.t.

a: Data represented as median ± mad (median absolute deviation) of 3-4 biologically independent experiments with 1 technical replicate each (n.t.: no translocation detected).
